# The Impact of Chronic Pancreatitis on the Occurrences of Human Cancers: Real-World Data

**DOI:** 10.3390/jcm12155102

**Published:** 2023-08-03

**Authors:** Chi-Chia Hsieh, Yi-Hsiu Fu, Nien-En Ku, Chia-Chun Hsia, Yu-Tung Hung, Tzu-Ju Hsu, Sung-Hsiung Chen, Shu-Jui Kuo

**Affiliations:** 1 Department of Education, Taipei Veterans General Hospital, Taipei 112201, Taiwan; chichia08160917@gmail.com; 2 Department of Education, Taichung Veterans General Hospital, Taichung 407219, Taiwan; yfu1103@gmail.com; 3 Department of Education, China Medical University Hospital, Taichung 404327, Taiwan; nicheerrr1007@gmail.com (N.-E.K.); jimmyhsia0422@gmail.com (C.-C.H.); 4 Management Office for Health Data, China Medical University Hospital, Taichung 404327, Taiwan; yutunghung1227@gmail.com (Y.-T.H.); r7r0923.cmuh@gmail.com (T.-J.H.); 5 Department of Orthopedic Surgery, College of Medicine, Chang Gung University, Kaohsiung Chang Gung Memorial Hospital, Kaohsiung 833401, Taiwan; 6 School of Medicine, China Medical University, Taichung 404328, Taiwan; 7 Department of Orthopedic Surgery, China Medical University Hospital, Taichung 404327, Taiwan

**Keywords:** chronic pancreatitis, pancreatic cancer, liver cancer, stomach cancer, Taiwanese National Health Insurance Database

## Abstract

Chronic pancreatitis (CP) may induce systemic inflammation, potentially increasing cancer susceptibility. However, the link between CP and extra-pancreatic cancer remains underexplored. Employing Taiwanese National Health Insurance Database data from 2000 to 2017, we compared 5394 CP patients with 21,576 non-CP individuals through propensity score matching. CP patients exhibited a significantly higher cancer risk (adjusted hazard ratio (aHR) of 1.32 for females and 1.68 for males) and cumulative incidence (*p* < 0.001) compared to non-CP individuals. CP showed notable associations with pancreatic (aHR = 3.51), liver (aHR = 1.62), stomach (aHR = 2.01), and other cancers (aHR = 2.09). In terms of liver cancer, CP was significantly associated with patients without viral hepatitis, regardless of gender (aHR = 2.01 for women; aHR = 1.54 for men). No significant cancer occurrences were observed within the first year following CP diagnosis. Pancreatic or liver cancer developed in approximately half of CP patients within 2–3 years, while gastric cancer in male CP patients predominantly occurred around the fifth year after diagnosis. These findings inform potential cancer-screening plans for CP patients.

## 1. Introduction

Chronic pancreatitis (CP) is a chronic inflammatory disease of the pancreas that could lead to irreversible damage to the pancreatic tissues and to associated exocrine and endocrine insufficiency [1,2,3,4,5]. The diagnosis of CP relies on a combination of image findings that indicate irreversible damage to the pancreas, such as calcification, stones, and duct stricture or dilation, along with manifested exocrine and endocrine dysfunctions [6]. 

Emerging evidence suggested that CP is not a localized disease confined to the pancreas [2]. CP is associated with elevated systemic levels of inflammatory mediators such as interleukin-6, tumor necrosis factor-α, interleukin-8, and members of the interleukin-1 family [7]. Systemic inflammation plays a significant role in the occurrence, staging, and progression of cancer [8]. Previous studies, albeit limited in sample size, have suggested a potential association between CP and the emergence of various extra-pancreatic cancers, comprising hepatic, pulmonary, colonic, and head and neck malignancies [9,10]. The association between CP, which is capable of inducing systemic inflammation, and cancer, a group of diseases associated with systemic inflammation, is thus intriguing and warrants further consideration.

CP can often be attributed to several prevalent risk factors, including excessive alcohol consumption, smoking, and genetic mutation [2,11,12,13]. Notably, alcohol use and smoking are well-established risk factors for various types of cancer. The shared risk factors, particularly alcohol and smoking, for CP and cancer provide additional grounds to consider the association between CP and cancer, in addition to the aforementioned factor of chronic inflammation. 

This dual association underscores the intricate and multifaceted nature of the interplay between CP, smoking, alcohol, and the susceptibility to different forms of cancer. Despite the potential for CP to predispose individuals to systemic complications, the available real-world data concerning the association between CP and extra-pancreatic cancers are limited and inconclusive. This dearth of epidemiological data on CP in the large-sample-size general population, particularly within the Han ethnic group, contributes to the uncertainty. Ethnic variations and discrepancies in case definitions may lead to disparate findings. The rationale behind our study was to investigate the correlation between CP and the prevalence of different types of cancer within the Taiwanese population. We hypothesized that CP patients would exhibit an elevated incidence of extra-pancreatic cancers.

## 2. Materials and Methods

This study utilized the Longitudinal Generation Tracking Database (LGTD 2005), which was derived from the Taiwanese National Health Insurance Database (NHIRD). The NHIRD comprises data collected through the National Health Insurance (NHI) program, a nationwide initiative implemented in Taiwan in 1995 to enhance the country’s healthcare and medical services. The LGTD encompasses demographic information of insured individuals, records of admissions and discharges, as well as medication and surgical procedure data for a randomly sampled population of 2 million Taiwanese individuals. To identify patients with cancer, we employed the linked registry of the Registry for Catastrophic Illness Patient Database (RCIPD), also derived from the NHIRD in Taiwan. Disease diagnoses were categorized utilizing the International Classification of Diseases, Ninth and Tenth Revision, and Clinical Modification (ICD-9-CM and ICD-10-CM) coding systems, as previously described in our published works [14,15,16,17,18,19,20,21,22,23,24,25]. Ethical approval for this study was obtained from the Institutional Review Board of the China Medical University Hospital Research Ethics Committee (CMUH109-REC2-031(CR-2)).

The study population included individuals aged 20 years or older but younger than 100 years of age, within the time frame of 1 January 2000, to 31 December 2017. These individuals were then categorized into two groups: those diagnosed with CP (identified by ICD-9 code 577.1 or ICD-10 code K86.1) and those without such a diagnosis. For patients diagnosed with CP, the index date was defined as the date of their initial CP diagnosis. For the control group, the index date was randomly assigned as the date when their follow-up period commenced after 1 January 2000. The two cohorts were matched based on sex, age (in 5-year intervals), year of the index date, Charlson Comorbidity Index (CCI), and NSAID (nonsteroidal anti-inflammatory drug) usage. Propensity score matching was employed with a 1:4 ratio. We excluded subjects who had a prior diagnosis of CP before the index date, individuals aged under 20 or above 100 years, those with missing sex or age data, and individuals with less than 1 year of follow-up time.

The primary objective of this study was to investigate the occurrence of various types of primary cancer as the study outcome. The types of cancer were classified as follows: liver (ICD-9: 155, ICD-10: C22), breast (ICD-9: 174, ICD-10: C50.0, C50.1, C50.2, C50.3, C50.411, C50.412, C50.419), lung (ICD-9: 162, ICD-10: C33, C34, C7A.090), thyroid (ICD-9: 193, ICD-10: C73, E31.22), colon rectum (ICD-9: 153,154, ICD-10: C18, C19, C20, C21, C7A.02), prostate (ICD-9: 185, ICD-10: C61), kidney (ICD-9: 189, ICD-10: C64, C65, C66, C68, C7A.093), nasopharynx (ICD-9: 147, ICD-10: C11), stomach (ICD-9: 151, ICD-10: C16, C7A.092), bladder (ICD-9: 188, ICD-10: C67), and other cancers [18]. The covariates considered in the analysis included sex, age categories (20–39, 40–59, ≥60), CCI, and NSIADs, which were compared between subjects with and without CP.

Categorical variables at baseline in different groups were evaluated using the chi-square test, whereas the means of continuous variables were compared using Student’s *t*-test. Incidence rates were calculated per 1000 person-years. The hazard ratios (HR) and their corresponding 95% confidence intervals (CIs) were estimated using the Cox proportional hazard regression model to assess the risk between the two cohorts. Both unadjusted (crude) hazard ratios (cHR) and multivariable-adjusted hazard ratios (aHR) were calculated. Subdistribution hazard ratios (SHR) and 95% CIs were determined using the competing-risk regression model. Cumulative incidence curves were constructed using the Kaplan–Meier method, and differences between the two cohorts were assessed using the log-rank test. Statistical analyses were performed using SAS software, version 9.4, and plots were generated using R software, version 4.0. The level of statistical significance was set at *p* < 0.05.

## 3. Results

Following propensity score matching, a total of 26,970 subjects were included in this study, with 5394 being CP patients and 21,576 being non-CP subjects. Table 1 presents the baseline characteristics and demographics of the participants, including gender, age, CCI score, and medication usage. Both groups had a higher proportion of male participants compared to female participants. There were no statistically significant differences in the distribution of CCI scores and NSAIDs between CP patients and the control group. The mean follow-up time for CP patients was 3.27 (±1.72) years, while for those without CP, it was 6.22 (±3.56) years.

**Figure 1:**
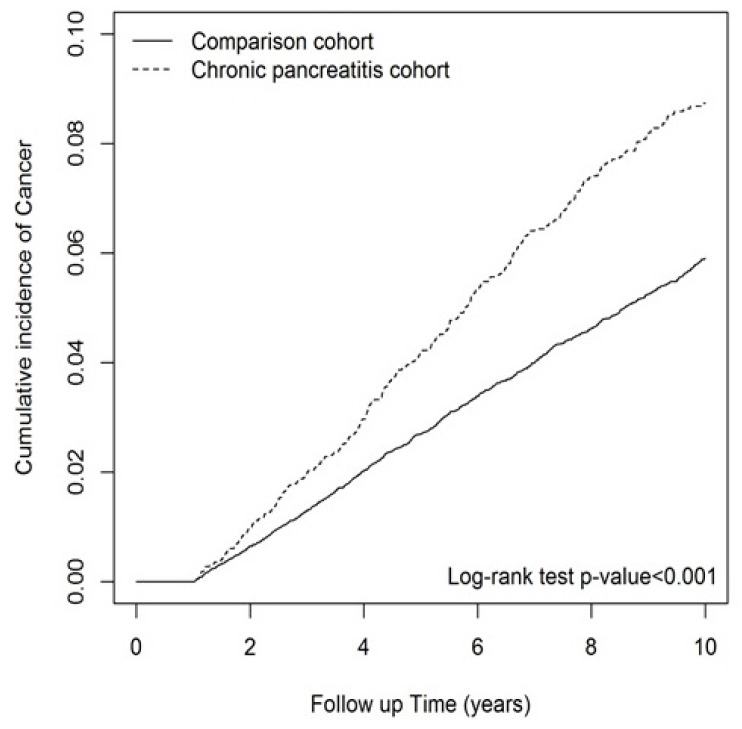
The cumulative incidence of all cancers in chronic pancreatitis and comparison cohorts.

Table 2 presents the risk of cancer in the two groups. After adjusting for age, sex, CCI score, and NSAID exposure, CP patients exhibited a higher risk of cancer (aHR = 1.58; 95% CI = 1.41, 1.77) compared to the control group. Moreover, the hazard ratio of cancer was higher in males (aHR = 1.43; 95% CI = 1.27, 1.60) than in females. Patients aged 40 to 59 or above 60 had a higher hazard ratio of cancer (40–59: aHR = 2.43; 95% CI = 2.07, 2.85/≥60: aHR = 5.18; 95% CI = 4.39, 6.12) compared to those aged 20 to 39. Regarding the CCI score and NSAIDs, individuals with higher CCI scores (1–2: aHR = 1.20; 95% CI = 1.07, 1.34/≥3: aHR = 1.75; 95% CI = 1.49, 2.05) and those taking NSAIDs (aHR = 1.62; 95% CI = 1.46, 1.80) had a higher risk of cancer. Table 2 also presents the adjusted SHR after considering death as a competing outcome. After adjusting for all covariates, there was a significantly higher risk of cancer in the CP group (adjusted SHR = 1.50; 95% CI: 1.34, 1.68). Males had a higher hazard ratio of cancer compared to females (adjusted SHR = 1.36; 95% CI: 1.20, 1.53). Patients aged 40 to 59 or above 60 had a higher risk of developing cancer than those aged 20 to 39 (40–59: adjusted SHR = 2.38; 95% CI = 2.03, 2.79/≥60: adjusted SHR = 4.33; 95% CI = 3.67, 5.12). Additionally, subjects with a CCI score above 3 (adjusted SHR = 1.25; 95% CI = 1.07, 1.47) and those taking NSAIDs (adjusted SHR = 1.61; 95% CI = 1.45, 1.79) had a higher risk of cancer. Figure 1 illustrates the cumulative incidence of cancer, which was significantly higher in the CP cohort compared to the non-CP cohort.

Table 3 presents the risk of cancer in individuals with CP compared to those without CP, stratified by sex and age. In both female and male patients, CP patients exhibited a significantly higher risk of cancer compared to the non-CP group (female: aHR = 1.32; 95% CI = 1.05, 1.66/male: aHR = 1.68; 95% CI = 1.47, 1.92). This increased risk was consistently observed across all age groups, with the CP cohort demonstrating higher risks compared to the controls (20–39: aHR = 2.38; 95% CI = 1.77, 3.19/40–59: aHR = 1.63; 95% CI = 1.38, 1.92/≥60: aHR = 1.32; 95% CI = 1.09, 1.59). Furthermore, CP patients had a higher risk of developing cancer compared to non-CP patients, regardless of their CCI score (CCI = 0: aHR = 1.57; 95% CI = 1.34, 1.83/CCI = 1–2: aHR = 1.51; 95% CI = 1.24, 1.83/CCI ≥ 3: aHR = 1.77; 95% CI = 1.30, 2.41). Similarly, a significantly elevated risk of cancer was observed for the CP cohort compared to the controls, regardless of NSAID use (non-NSAIDs: aHR = 1.80; 95% CI = 1.50, 2.15/NSAIDs: aHR = 1.45; 95% CI = 1.25, 1.68). In other words, CP patients were consistently associated with a higher risk of cancer, irrespective of sex, age, CCI score, and NSAID exposure. 

Table 4 provides a stratification of primary cancer locations by gender. Among men, individuals with CP exhibited a significantly higher risk of stomach cancer (aHR = 2.19; 95% CI = 1.02, 4.68) and other cancers (aHR = 2.45; 95% CI = 2.03, 2.96). Furthermore, irrespective of gender, CP patients had elevated risks of liver cancer (women: aHR = 2.01; 95% CI = 1.09, 3.72/men: aHR = 1.54; 95% CI = 1.06, 3.79) and pancreatic cancer (women: aHR = 16.20; 95% CI = 1.46, 179.70/men: aHR = 3.17; 95% CI = 1.12, 9.04) compared to the non-CP group.

Table 5 presents the stratified risk of liver cancer based on viral carrier status and sex. Notably, among males without hepatitis B virus (HBV) and hepatitis C virus (HCV) infections, CP patients demonstrated a significantly elevated risk of liver cancer (aHR = 1.49; 95% CI = 1.02, 2.18) compared to non-CP individuals.

**Figure 2:**
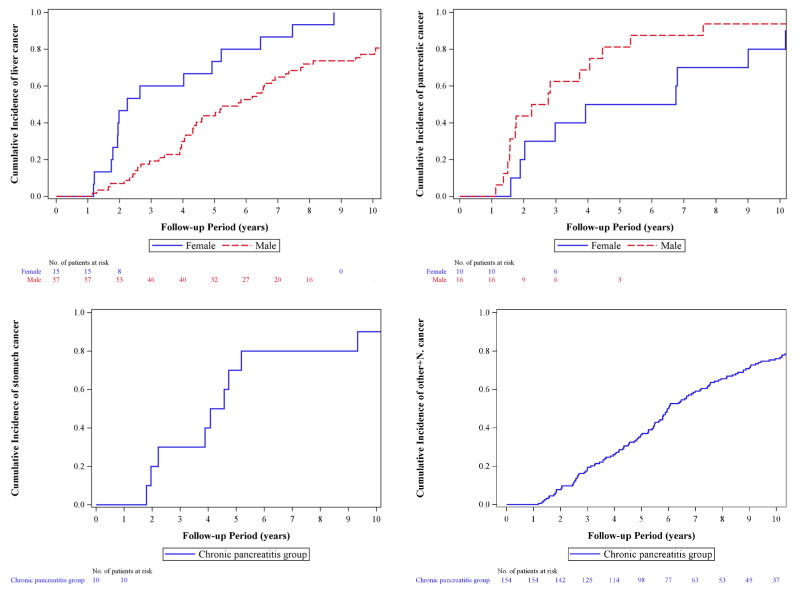
Timeline of liver cancer, pancreatic cancer, gastric cancer, and other cancers, including nasopharyngeal cancer, occurring within a ten-year follow-up period in CP patients. To comply with government data security regulations, patient information with values or differences below three has been excluded from the chart.

Figure 2 provides an overview of the temporal association between liver cancer, pancreatic cancer, stomach cancer, and other cancers, including nasopharyngeal cancer diagnoses, in patients with CP. Remarkably, no occurrences of these cancers were observed within the first year following a CP diagnosis in patients. Subsequently, among male and female CP patients who developed pancreatic cancer, as well as female CP patients who developed liver cancer, close to half of the patients experienced cancer onset within 2–3 years after the CP diagnosis. In male CP patients who developed gastric cancer, approximately half of the cases occurred during the fifth year following the CP diagnosis.

## 4. Discussion

Alcohol abuse and smoking are common risk factors shared by both CP and various types of cancer. Therefore, it is reasonable to make hypotheses about the potential association between CP and the development of human cancers. Our study revealed a significant correlation between CP and the development of various human cancers, notably liver and stomach cancer. By leveraging a nationwide registry database and encompassing a substantial sample size, we conducted this investigation within the Taiwanese population, allowing us to gather robust real-world data pertaining to the impact of CP on cancer risk. The study employed a longitudinal design, spanning a 17-year period, and included a cohort of approximately 2,000,000 residents of Taiwan. The extended duration and large sample size ensured high statistical power and minimized selection bias. Notably, the NHI program covers over 99.9% of Taiwan’s population, enhancing the generalizability of our findings to the entire populace. These findings offer compelling evidence that CP manifests systemic effects beyond its localized nature. Recognizing the public health implications, future research should concentrate on evaluating the efficacy of cancer screening protocols tailored specifically for CP patients. This discovery opens up promising avenues for cancer prevention strategies and carries significant implications for clinical practice and future research endeavors in this domain.

The precise mechanisms underlying the development of cancer in patients with CP remain unclear. Various potential mechanisms have been proposed, with systemic chronic inflammation emerging as a potential factor. CP represents an infectious process that, if not adequately addressed, can lead to systemic chronic inflammation. Inflammatory processes can generate free radicals and active oxidative/nitrosative intermediates, which could contribute to DNA mutations and interfere with DNA repair mechanisms within cells [26]. Moreover, inflammatory cells themselves may perpetuate the cycle by producing free radicals, cytokines, chemokines, and arachidonic acid metabolites, leading to the recruitment of more inflammatory cells [26]. Despite the demonstrated potential of specific anti-inflammatory drugs in preventing or mitigating the risk of certain cancers at different anatomical sites (e.g., colorectal, esophageal, gastric, biliary tract, and breast cancers), our study produced contrasting findings. One possible explanation for this discrepancy is that patients with higher CP severity may consume larger quantities of NSAIDs, thereby making NSAID exposure correlate with CP severity. 

In our study, despite having only 41 cases of pancreatic cancer in our population, we observed a significant association between CP and pancreatic cancer in both sexes. This result is consistent with the findings of other studies conducted to date, indicating that CP is a risk factor for pancreatic cancer [27]. This further strengthens the credibility of our data.

Hepatitis B and C viruses have been shown to cause acute and chronic infections that lead to the development of hepatocellular carcinoma, which accounts for 90% of all liver cancer [28,29]. In our study, male CP patients without hepatitis B or C were found to have a higher risk of liver cancer. This discovery supports the notion that CP is an independent risk factor for liver cancer, which may be caused by the systemic inflammation associated with CP.

Some studies have shown no association between CP and stomach cancer; our study discovered a significant association between CP and stomach cancer. However, CP and stomach cancer share similar risk factors, such as alcohol use and smoking [30]. Our study failed to retrieve information concerning alcohol use and smoking. We hypothesize that the results might be different after modifying the factors of alcohol use and smoking. 

In one Taiwanese population-based study, it was found that CP is a risk factor for head and neck cancer, and the risk increases with comorbidity [10]. In our study, we observed a comparable pattern and noted a more pronounced influence of CP on head and neck cancer (shown in “other” cancer). The differential outcomes between genders could potentially be explained by the existence of shared risk factors linking CP and head and neck cancer, which are more prevalent among Taiwanese males. Factors such as smoking, alcohol consumption, and betel nut chewing are more frequently observed in males within Taiwanese society and are recognized as established risk factors for head and neck cancer [31].

Table 6 provides a summary of the previous research that has investigated the association between CP and cancer. Two studies found that CP patients had a higher risk of liver cancer, which is consistent with our findings. One study indicated that the risk of liver cancer decreased after omitting liver cancer cases diagnosed within a year of admission for pancreatitis [32]. One possible explanation is that when patients are diagnosed with pancreatitis, concurrent liver cancer is also detected. Another cohort study by Agarwal S et al. examined the risk of pancreatic cancer among patients with different types of chronic pancreatitis, including alcoholic chronic pancreatitis (ACP), idiopathic juvenile chronic pancreatitis (IJCP), and idiopathic senile chronic pancreatitis (ISCP). The study found that ISCP was associated with a higher risk of pancreatic cancer [33]. 

Our study revealed that no significant occurrences of cancers were observed within the first year following CP diagnosis. Among CP patients who developed pancreatic cancer (both sexes) and female CP patients with liver cancer, nearly half experienced cancer onset within 2–3 years after the CP diagnosis. For male CP patients with gastric cancer, approximately half of the cases occurred during the fifth year after the CP diagnosis. Notably, the intensity of CP follow-up decreased over time in the clinical setting. Our study established that the “risk window” opens at least one year after CP diagnosis and persists for up to five years. Consequently, we recommend rigorous cancer screening for CP patients between one and five years after CP diagnosis. During the initial five years following the diagnosis of CP, it is imperative for both clinicians and patients to approach the condition with vigilance.

This study’s primary limitation is the lack of lifestyle information in the utilized database, particularly concerning patients’ alcohol consumption and smoking habits. However, it is noteworthy that lung cancer consistently exhibits the highest hazard ratio among smokers in different studies [42,43], and esophageal cancer is most strongly associated with alcohol consumption [44,45]. In our cohort, neither lung nor esophageal cancer showed a significant association with CP. Consequently, the observed CP–cancer association cannot be solely attributed to alcohol consumption or smoking. Moreover, important patient data, including genetic predisposition, family history, and environmental influences on tumor growth, were not available in NHIRD. Additionally, surveillance bias may influence our findings, as CP-diagnosed individuals often undergo regular monitoring, leading to increased exposure to cancer screenings and referrals, potentially impacting the observed outcomes. Our research design does not definitively elucidate the underlying mechanisms driving the CP–cancer association. In spite of these limitations, our study provides supportive evidence for a robust association between CP and human cancer. To gain a more comprehensive understanding, future studies should address these limitations and explore potential mechanistic links. 

## 5. Conclusions

Our population-based cohort study demonstrated a significant correlation between CP and heightened susceptibility to specific types of cancer in humans, most notably liver, pancreatic, and stomach cancer. However, to establish a concrete association and gain deeper insights into the underlying pathogenesis linking CP to human cancer, further investigations in the form of randomized controlled prospective studies and clinical trials are imperative. The development of a screening program tailored to CP patients should be considered to enable the early detection of cancer. This proactive approach holds the potential to significantly improve patient outcomes. We firmly believe that our findings can contribute to the implementation of appropriate strategies for the follow-up care for CP patients, while also raising public awareness regarding the heightened cancer risk associated with this condition.

## Figures and Tables

**Table 1:** Comparison of demographic characteristics and comorbidities between patients with and without chronic pancreatitis.

	CP (−)		CP (+)		*p* Value
	N	%	N	%	
Gender					0.979
Female	6876	31.87	1720	31.89	
Male	14,700	68.13	3674	68.11	
Age					0.213
20~39	5765	26.72	1504	27.88	
40~59	10,251	47.51	2534	46.98	
60~	5560	25.77	1356	25.14	
Mean (SD)	50.09	(14.93)	49.71	(14.96)	0.101
CCI score					0.999
0	12,367	57.32	3091	57.30	
1~2	7029	32.58	1757	32.57	
3~	2180	10.10	546	10.12	
NSAIDs					0.733
No	11,084	51.37	2785	51.63	
Yes	10,492	48.63	2609	48.37	
Mean (SD) follow-up years	6.22	(3.56)	3.27	(1.72)	0.021

CP: chronic pancreatitis; SD: standard deviation; CCI score: Charlson comorbidity index score; NSAIDs: non-steroid anti-inflammatory drugs.

**Table 2:** The crude, adjusted, and subdistribution hazard ratios of all cancers stratified by the presence of chronic pancreatitis, gender, age, Charlson comorbidity index score, and use of non-steroid anti-inflammatory drugs.

	All Cancer			Crude			Adjusted			Adjusted		
	N	PYs	IR	cHR	95% CI	*p* Value	aHR	95% CI	*p* Value	aSHR	95% CI	*p* Value
CP												
No	1108	188,156.6	5.89	1.00	reference	-	1.00	reference	-	1.00	reference	-
Yes	405	44,363.2	9.13	1.56	1.39~1.74	<0.001	1.58	1.41~1.77	<0.001	1.50	1.34~1.68	<0.001
Gender												
Female	401	71,620.3	5.60	1.00	reference	-	1.00	reference	-	1.00	reference	-
Male	1112	160,899.5	6.91	1.23	1.09~1.38	<0.001	1.43	1.27~1.60	<0.001	1.36	1.20~1.53	<0.001
Age (year)												
20~39	191	71,719.1	2.66	1.00	reference	-	1.00	reference	-	1.00	reference	-
40~59	721	112,061.8	6.43	2.44	2.08~2.86	<0.001	2.43	2.07~2.85	<0.001	2.38	2.03~2.79	<0.001
60~	601	48,738.9	12.33	4.84	4.11~5.70	<0.001	5.18	4.39~6.12	<0.001	4.33	3.67~5.12	<0.001
CCI score												
0	782	144,026.0	5.43	1.00	reference	-	1.00	reference	-	1.00	reference	-
1~2	526	71,548.0	7.35	1.36	1.22~1.52	<0.001	1.20	1.07~1.34	0.001	1.09	0.98~1.23	0.118
3+	205	16,945.7	12.10	2.34	2.00~2.73	<0.001	1.75	1.49~2.05	<0.001	1.25	1.07~1.47	0.006
NSAIDs												
No	582	118,873.8	4.90	1.00	reference	-	1.00	reference	-	1.00	reference	-
Yes	931	113,646.0	8.19	1.67	1.51~1.86	<0.001	1.62	1.46~1.80	<0.001	1.61	1.45~1.79	<0.001

PYs: person-years; IR: incidence rate; CI: confidence interval; cHR: crude hazard ratio; aHR: adjusted hazard ratio; aSHR: adjusted subdistribution hazard ratio; CCI score: Charlson comorbidity index score; NSAIDs: non-steroid anti-inflammatory drugs.

**Table 3:** The crude and adjusted hazard ratios of all cancers among the subjects with and without chronic pancreatitis stratified by gender, age, Charlson comorbidity index score, and the use of non-steroid anti-inflammatory drugs.

All Cancers												
	CP (−)			CP (+)			Crude			Adjusted		
	N	PYs	IR	N	PYs	IR	cHR	95% CI	*p*-Value	aHR	95% CI	*p*-Value
Gender												
Female	304	57,596.21	5.28	97	14,024.08	6.92	1.31	1.05~1.65	0.019	1.32	1.05~1.66	0.017
Male	804	130,560.4	6.16	308	30,339.12	10.15	1.66	1.45~1.89	<0.001	1.68	1.47~1.92	<0.001
Age (year)												
20~39	121	57,679.12	2.10	70	14,039.95	4.99	2.42	1.81~3.25	<0.001	2.38	1.77~3.19	<0.001
40~59	528	91,100.87	5.80	193	20,960.93	9.21	1.60	1.35~1.88	<0.001	1.63	1.38~1.92	<0.001
60~	459	39,376.59	11.66	142	9362.32	15.17	1.31	1.08~1.58	0.006	1.32	1.09~1.59	0.004
CCI score												
0	568	116,324.3	4.88	214	27,701.77	7.73	1.59	1.36~1.86	<0.001	1.57	1.34~1.83	<0.001
1~2	392	58,042.34	6.75	134	13,505.67	9.92	1.48	1.22~1.81	<0.001	1.51	1.24~1.83	<0.001
3+	148	13,789.97	10.73	57	3155.76	18.06	1.71	1.26~2.32	<0.001	1.77	1.30~2.41	<0.001
NSAIDs												
No	410	96,081.71	4.27	172	22,792.05	7.55	1.77	1.48~2.12	<0.001	1.80	1.50~2.15	<0.001
Yes	698	92,074.86	7.58	233	21,571.15	10.80	1.43	1.24~1.66	<0.001	1.45	1.25~1.68	<0.001

PYs: person-years; IR: incidence rate; CI: confidence interval; cHR: crude hazard ratio; aHR: adjusted hazard ratio; aSHR: adjusted subdistribution hazard ratio; CCI score: Charlson comorbidity index score; NSAIDs: non-steroid anti-inflammatory drugs.

**Table 4:** The crude and adjusted incidence rates of various cancers for the subjects with and without chronic pancreatitis.

	CP (−)			CP (+)			Crude			Adjusted		
	N	PYs	IR	N	PYs	IR	cHR	95% CI	*p*-Value	aHR	95% CI	*p*-Value
Female												
Liver	32	55,954.01	0.57	15	13,552.22	1.11	1.94	1.05~3.59	0.034	2.01	1.09~3.72	0.025
Breast	17	55,797.93	0.30	6	13,532.12	0.44	1.45	0.57~3.67	0.435	1.45	0.57~3.68	0.434
Lung	28	55,941.09	0.50	9	13,561.11	0.66	1.34	0.63~2.83	0.448	1.33	0.63~2.81	0.462
Thyroid	13	55,807.78	0.23	4	13,522.32	0.30	1.27	0.41~3.89	0.678	1.28	0.42~3.92	0.667
Colorectal	44	55,992.41	0.79	10	13,566.08	0.74	0.94	0.47~1.86	0.856	0.94	0.47~1.87	0.859
Kidney + Bladder	21	55,861.04	0.38	4	13,512.90	0.30	0.79	0.27~2.29	0.662	0.80	0.27~2.33	0.681
Stomach	10	55,783.60	0.18	4	13,525.42	0.30	1.65	0.52~5.27	0.397	1.66	0.52~5.30	0.390
Pancreatic	3	25.25	118.83	10	56.66	176.49	2.31	0.50~10.67	0.285	16.20	1.46~179.70	0.023
Other + NP	136	56,585.40	2.40	35	13,686.26	2.56	1.07	0.73~1.54	0.739	1.07	0.74~1.55	0.732
Male												
Liver	167	126,522.20	1.320	57	28,791.8	1.98	1.50	1.11~2.03	0.008	1.54	1.14~2.08	0.005
Lung	88	126,131.09	0.698	21	28,541.08	0.74	1.05	0.66~1.70	0.826	1.05	0.65~1.68	0.855
Thyroid	8	125,666.5	0.064	3	28,440.52	0.11	1.67	0.44~6.31	0.447	1.69	0.45~6.36	0.440
Colorectal	104	126,271.97	0.824	24	28,555.55	0.84	1.03	0.66~1.60	0.904	1.03	0.66~1.61	0.882
Prostate	66	126,027.67	0.524	12	28,502.86	0.42	0.81	0.44~1.51	0.512	0.77	0.41~1.42	0.400
Kidney + Bladder	46	125,852.34	0.366	11	28,478.92	0.39	1.05	0.54~2.03	0.887	1.07	0.55~2.06	0.845
Stomach	20	125,732.778	0.159	10	28,477.04	0.35	2.21	1.04~4.73	0.040	2.19	1.02~4.68	0.043
Pancreatic	12	80.55	148.97	16	56.08	285.30	2.35	1.06~5.22	0.036	3.17	1.12~9.04	0.031
Other + NP	293	127,539.21	2.30	154	29497.62	5.22	2.29	1.88~2.79	<0.001	2.32	1.90~2.82	<0.001

PYs: person-years; NP: nasopharyngeal.

**Table 5:** The crude and hazard ratios of liver cancer among patients with and without CP, stratified by the presence of virus carrier state.

Liver Cancer												
	CP (−)			CP (+)			Crude			Adjusted		
	N	PYs	IR	N	PYs	IR	cHR	95% CI	*p* Value	aHR	95% CI	*p* Value
HBV (−)/HCV (−)	120	170,746.88	0.70	47	38,717.57	1.21	1.74	1.25~2.44	0.001	1.40	0.99~1.99	0.058
HBV (−)/HCV (+)	36	3022.88	11.91	13	994.81	13.07	1.11	0.59~2.09	0.755	1.08	0.56~2.05	0.824
HBV (+)/HCV (−)	38	7830.48	4.85	12	2631.64	4.33	0.90	0.45~1.80	0.761	0.87	0.43~1.76	0.695
HBV (+)/HCV (+)	5	875.97	5.71			6.21	1.14	0.22~5.86	0.879	1.40	0.24~8.16	0.705
(**a**) The crude and hazard ratios of liver cancer among female patients with and without CP, stratified by the presence of virus carrier state.												
**Liver cancer (female CP patients)**												
	**CP (−)**			**CP (+)**			**Crude**			**Adjusted**		
	**N**	**PYs**	**IR**	**N**	**PYs**	**IR**	**cHR**	**95% CI**	***p *** **Value**	**aHR**	**95% CI**	***p *** **Value**
HBV (−)/HCV (−)	23	53,672.81	0.43	6	12,729.65	0.47	1.11	0.45~2.72	0.821	1.07	0.43~2.65	0.881
HBV (−)/HCV (+)	10	2281.21	11.42	9	822.57	21.69	1.85	0.67~5.12	0.234	1.81	0.65~5.10	0.259
HBV (+)/HCV (−)			0.69			2.34						
HBV (+)/HCV (+)			7.84			13.64	2.34	0.14~37.93	0.550	4.29	0.10~185.28	0.449
(**b**) The crude and hazard ratios of liver cancer among male patients with and without CP, stratified by the presence of virus carrier state.												
**Liver cancer (male CP patients)**												
	**CP (−)**			**CP (+)**			**Crude**			**Adjusted**		
	**N**	**PYs**	**IR**	**N**	**PYs**	**IR**	**cHR**	**95% CI**	***p *** **Value**	**aHR**	**95% CI**	***p *** **Value**
HBV (−)/HCV (−)	97	117,074.08	0.83	41	25,987.91	1.58	1.92	1.33~2.77	<0.001	1.49	1.02~2.18	0.040
HBV (−)/HCV (+)	28	2322.35	12.06	6	672.07	8.93	0.77	0.32~1.86	0.562	0.74	0.30~1.83	0.513
HBV (+)/HCV (−)	38	6377.32	5.96	10	2131.81	4.78	0.81	0.39~1.67	0.563	0.77	0.37~1.61	0.495
HBV (+)/HCV (+)	4	748.45	5.34			4.02	0.75	0.08~6.75	0.801	0.26	0.02~4.41	0.351

PYs: person-years; IR: incidence rate; cHR: crude hazard ratio; aHR: adjusted hazard ratio; CI: confidence interval.

**Table 6:** Previous publications on the association between CP and cancer occurrence.

	Design	Population Characteristic	Cancer Type	Outcome
Bang UC, et al. (2014) [34]	Cohort	Data source: Danish National Patient Register (1995–2010) Papulation/Control: 11,972/119,720	All cancers	All cancers: 13.6% CP cases experienced a cancer compared with 7.9% of the controls (*p* < 0.0001), aHR of 1.2 (95% CI, 1.1–1.3). Pancreas and Liver cancer: CP cases had significantly higher risks with aHRs of 6.9 (95% CI, 5.6–8.6) and 2.0 (95% CI, 1.3–3.1) for pancreatic and liver cancer, respectively. The RR of pancreatic cancer was particularly elevated during the initial 2–4 years after cohort entry (adjusted HR, 14.6; 95% CI, 10.9–19.6)
Goldacre MJ, et al. (2008) [32]	Cohort	Data source: Oxford record linkage study (ORLS)(1963–1999). Papulation/Control: 1496/599,308	All cancers	All cancers: The RRs for cancer overall were 1.3 (1.1–1.4) AP patients and 2.5 (2.1–2.8) in CP patients. Liver cancer: The RRs were 2.3 (95% CI, 1.3–4.0) for AP and 5.6 (95% CI, 2.1–12.4) for CP. However, after excluding liver cancer cases diagnosed within one year of pancreatitis admission, the association was not significant. The RRs were 3.0 (95% CI, 2.2–4.0) for AP and 10.7 (95% CI, 7.3–15.3) for CP. Pancreatic cancer: The RRs were 5.7 (4.5–7.1) associated with AP and 27.0 (21.4–33.8) associated with CP; they dropped over time, but remained significantly high after omitting the first year cases: they were 3.0 (2.2–4.0) in AP and 10.7 (7.3–15.3) in CP. Lung cancer: Significantly high in people with AP (RRs: 1.3; 1.0–1.6) and in CP (RRs: 2.3; 1.5–3.3).
Chen CH, et al.(2020) [10]	Cohort	Data source: Taiwan National Health Insurance (NHI) program (2000–2011) Papulation/Control: 11,237/11,237	Head and Neck cancer	Head and neck cancer: Patients with CP had a significantly higher risk of head and neck cancer (aHR = 1.31, 95% CI: 1.07–1.60) and a greater incidence of head and neck cancer (log-rank test, *p* < 0.001). The incidence of head and neck cancer in the CP cohort was 1.90%, while in the non-CP cohort, it was 1.60%. This represents a 0.30% absolute risk increase.
Karlson BM, et al. (1997) [35]	Cohort	Data source: Swedish Inpatient Registry (1965–1983) Papulation: 29,530 Sub-cohorts: (1) one episode of unspecified pancreatitis (n = 823); (2) one episode of acute pancreatitis (*n* = 24,753); (3) recurrent pancreatitis (*n* = 7328); (4) chronic pancreatitis (*n* = 4546).	Pancreatic cancer	Excess risks for pancreatic cancer were observed in all sub-cohorts. The SIR for all cohorts combined was 2.8 (95% CI, 2.5–3.2). The highest risks were observed in the patients with CP (SIR, 7.6; 95% CI, 6.0–9.7) and with one attack of unspecified pancreatitis (SIR, 7.3; 95% CI, 3.5–13.4).
Anderson LN, et al. (2009) [36]	Case-control	Data source: Ontario Cancer Registry (2003–2007) Case/control: 422/413	Pancreatic cancer	CP was found to be associated with increased risk in the age-adjusted model, yet after adjustment for other variables, this association approached null and was not significant.
Bansal and Sonnenberg et al. (1995) [37]	Case-control	Data source: Veterans Affairs (VA) Case/control: 2639/7774	Pancreatic cancer	In a multivariate analysis, the odds ratios for all types of pancreatitis and CP alone were 3.42 (CI, 1.98–5.91) and 2.23 (CI, 1.43–3.49), respectively.
Jeon CY, et al. (2020) [38]	Cohort	Data source: healthcare system in Southern California (2006–2015). Case: 1766	Pancreatic cancer	Obesity (HR, 2.7, 95% CI: 1.2–6.1) and duct dilatation (HR, 10.5, 95% CI: 4.0–27) were identified as predictive factors for incident pancreatic cancer after 1 year of follow-up. The five-year incidence of pancreatic cancer in patients with duct dilatation was 6.3%.
Hao L, et al. (2017) [39]	Cohort	Data source: Changhai Hospital center (2000–2013) Case: 1656	Pancreatic cancer	Of a total of 1656 patients, the median follow-up duration was 8.0 years. Pancreatic cancer was detected in 21 patients (1.3%). The expected number of cases of pancreatic cancer was 1.039, yielding an SIR of 20.22.
Lowenfels AB, et al. (1993) [40]	Cohort	Data source: Multiple clinical centers in six countries. Case: 2015	Pancreatic cancer	The risk of developing pancreatic cancer steadily increased over time. At 2 years and 20 years after the diagnosis of pancreatitis, the cumulative risks were 1.8% (95% CI: 1.0 to 2.6%) and 4.0% (95% CI: 2.0 to 5.9%), respectively.
Agarwal S, et al. (2020) [33]	Cohort	Data source: A tertiary care center at India (1998 -2019) Case: 1415	Pancreatic cancer	Ten-year risk of pancreatic cancer was 0.9%, 0.2% and 5.2% in alcoholic pancreatitis, idiopathic-juvenile chronic pancreatitis and idiopathic senile chronic pancreatitis, respectively.
Midha S, et al. (2016) [41]	Cohort/ Case-control	Data source: Tertiary care academic center. Case/Control: 402 in cohort study 249/1000 in case-control study	Pancreatic cancer	Cohort study: During 3967.74 person-years of exposure, 5 of the 402 patients (4 idiopathic CP, 1 hereditary CP) developed pancreatic cancer after 16.60 ± 3.51 years of CP. The SIR was 121. Case-control study: Multivariable analysis showed CP (OR, 97.67; 95% CI, 12.69–751.36), diabetes (>4 years duration) (OR, 3.05; 95% CI, 1.79–5.18), and smoking (OR, 1.93; 95% CI, 1.38–2.69) as significant risk factors for pancreatic cancer.

AP: acute pancreatitis; CP: chronic pancreatitis; CI: confidence interval; HR: hazard ratio; RR: rate ratio; SIR: standardized incidence ratio.

## Data Availability

The data presented in this study are available on request from the corresponding author.
